# Impact of a Pharmacist-Led HCV Treatment Program at a Federally Qualified Health Center

**DOI:** 10.3390/pharmacy12040115

**Published:** 2024-07-24

**Authors:** Jerika T. Lam, Sharon Xavioer

**Affiliations:** Department of Pharmacy Practice, Chapman University School of Pharmacy, 9401 Jeronimo Rd. Ste 207, Ste 296, Irvine, CA 92618, USA; xavioer@chapman.edu

**Keywords:** hepatitis C virus, direct-acting antivirals, pharmacists, HCV/HIV co-infection, sustained virologic response, FQHC

## Abstract

Pharmacists are key players who can help to eliminate the hepatitis C virus (HCV) epidemic in the United States. This pilot retrospective study evaluated the impact of a pharmacist-led HCV treatment program in a federally qualified health center (FQHC) primary care clinic setting. The primary outcome was to assess sustained virologic response (SVR) rates 12 weeks after patients were initiated and completed their oral direct acting antiviral (DAA) treatment regimens. Methods: This pilot retrospective study included historical analyses of patients who received DAA treatment in the pharmacist-led HCV treatment program in a FQHC clinic between 1 January 2019 and 31 January 2021. SVR was the primary outcome measure for treatment response. Results: Sixty-seven patients with HCV mono- and HIV co-infection were referred, and 59 patients were initiated on DAA regimens after treatment. Fifty of those who were started on DAA regimens completed their treatment, and 38 achieved SVR (modified intention to treat [mITT] SVR rate of 76%). Conclusion: Our study’s findings demonstrated SVR rates that were comparable with other pharmacist-directed HCV treatment services in the United States despite the impact of the COVID-19 pandemic. Our study included a higher proportion of individuals with HCV/HIV co-infection and of Hispanic ethnicity.

## 1. Introduction

Chronic hepatitis C virus (HCV) infection is a major public health issue. It affects an estimated 2.4 to 3 million people in the United States (US) [1,2], with 107,300 new HCV cases recorded in 2020 alone [3]. While some people may spontaneously clear the virus, more than half will develop chronic infection. Approximately 5 to 25% of infected individuals will develop cirrhosis within 10 to 20 years, and 1 to 5% will die from complications related to cirrhosis or hepatocellular carcinoma. HCV infection remains a leading cause of liver transplantation [4]. Due to the notable burden that HCV places on public health, the World Health Organization (WHO) has proposed a goal to reduce new chronic infections by 90% and liver-associated mortality by 65% by 2030 [4,5]. As a result, the Department of Health and Human Services has created the Viral Hepatitis National Strategic Plan for the United States effective through 2025. This roadmap calls for the expansion of HCV screening and treatment and specifically promotes the inclusion of pharmacists in testing, counseling, and treatment management [6].

The arrival of oral direct-acting antivirals (DAAs) in 2011 drastically changed the landscape of HCV treatment. Previous treatment options were limited to injectable interferons and oral ribavirin that led to significant adverse effects, low cure rates, and long treatment durations of up to one year [7,8]. In contrast, oral DAA medications have simplified the treatment regimens, shortened treatment durations of between 8 and 12 weeks, improved tolerability, and achieved cure rates of more than 95%. Despite these significant advancements, chronic HCV infection continues to pose a threat, especially due to continued intravenous drug use among persons aged 18–40 years old [1]. People with HCV infection face several barriers to treatment access including HCV stigma, asymptomatic infections leading to delayed or missed diagnosis, limited knowledge regarding HCV, and more [8]. Those within underserved and marginalized populations may face additional barriers including access to medications and linkage to care.

Prior studies have evaluated the impact of specialist- and non-specialist-led HCV treatment services for underserved populations within the federally qualified health center (FQHC) setting [9,10,11]. These studies have shown the positive impact of proactive HCV treatment by achieving high sustained virologic response (SVR) rates or virologic cures. Very few studies have assessed the impact of pharmacist-led HCV treatment programs [9,11,12]. Programs that completely integrate pharmacists have achieved comparable or high SVR rates. There continues to be gaps, however, in understanding the impact that pharmacists have in providing HCV treatment services within underserved, marginalized populations. We also have limited knowledge of how pharmacists can assist patients with other risk factors, such as co-infection with HIV, and help them to successfully complete treatment. Greater understanding of these factors could demonstrate how pharmacists can be leveraged as key players in the HCV treatment cascade to help the US to achieve the national and international goal of HCV infection elimination.

To address the current HCV public health crisis in the US, this pilot retrospective study evaluated the impact of a pharmacist-led HCV treatment program for people with HCV mono- and HCV/HIV co-infections at an FQHC primary care clinic setting. The primary outcome of the pilot study was to evaluate SVR rates 12 weeks after patients completed their DAA treatment regimens in the program.

## 2. Methods

This was a pilot retrospective study that included historical analyses of patients who received HCV treatment in the pharmacist-led HCV treatment program at AltaMed clinic between 1 January 2019 and 31 January 2021. This study received institutional review board approval from Chapman University in collaboration with AltaMed Health Services.

### 2.1. Study Setting and Participants

AltaMed Health Services is an FQHC that provides medical care for low-income and underserved people in southern California. This study was conducted at an AltaMed clinic site located in Anaheim, Orange County, CA, USA. The majority of the study patients were underserved and contracted HCV infection from intravenous drug use. Clinical pharmacy services were provided by two advanced practice ambulatory care pharmacists, who were also faculty members at the Chapman University School of Pharmacy. The interdisciplinary team included the pharmacists and medical providers from internal medicine, infectious disease, and family practice providers. The referral process, DAA treatment protocol, and follow-up care were adopted from existing national guidance for standards of care [13]. The HCV treatment workflow process began after the patient was diagnosed by a physician or nurse practitioner and referred to the pharmacists for HCV education and treatment management in the EPIC communication portal. Working under a collaborative practice agreement with AltaMed, the ambulatory care pharmacists initiated DAA regimens, screened patients’ concomitant medications for drug–drug interactions, ensured that the DAA regimens were covered by patients’ health insurance, ordered and monitored laboratory test values, provided adherence counseling, and scheduled patients for follow-up care in the clinic or via telehealth visits.

### 2.2. Patient Characteristics

Eligible patients were adults aged 18 years and older, had a confirmed HCV antibody and RNA viral load, and were referred by one of the medical providers at the AltaMed clinic in Anaheim. Patients were excluded from the analysis if they continued their DAA treatments after 31 January 2021, were pregnant, or did not receive DAA treatment and management from one of the ambulatory care pharmacists. Other patient characteristics that were included in the analysis were age, gender, ethnicity, insurance type, coexisting health conditions (e.g., diabetes mellitus, dyslipidemia, hypertension), HCV genotype, HCV RNA viral load, liver fibrosis stage, HIV co-infection status, injection drug use history, baseline laboratory biomarkers (e.g., complete blood count, platelets, hepatic transaminases, serum creatinine, protime/INR), and concomitant medications. Liver fibrosis staging was determined using a noninvasive blood test called Fibrosure. The following thresholds for liver fibrosis staging were used: <0.5 for F0–F1, 0.5–1.5 for F2–F3, and >1.5 for F4 [14].

Sustained virologic response (SVR) or HCV cure was the primary outcome measure, which is defined as having an undetectable HCV RNA viral load (less than 15 IU/mL) at least 12 weeks after treatment completion. Secondary outcomes included baseline on-treatment side effects and drug–drug interactions. A modified intent-to-treat (mITT) or per-protocol analysis approach was used in which patients who discontinued therapy or were lost to follow-up during the study period were excluded. Patients identified as lost to follow-up care were those who did not have an SVR result available at least 12 weeks after treatment completion, discontinued their treatment after the first month of treatment, or were incarcerated. Data were analyzed using GraphPad Prism 9.0 software (San Diego, CA, USA). Descriptive statistics were performed to examine associations between the study variables using a paired t-test. Statistical significance was defined at a *p* value < 0.05.

## 3. Results

Between 1 January 2019 and 31 January 2021, 67 patients with HCV mono- and HIV co-infection were referred (“enrolled”) to the pharmacist-led HCV treatment program. There were 39 patients referred to the program in 2019, 23 patients in 2020, and 5 patients in 2021. Fifty-nine patients were initiated on DAA regimens after treatment discussions with the pharmacists and screening for significant drug–drug interactions with their concomitant medications. In total, 50 of those who were started on DAA regimens completed their treatment, and 38 achieved SVR or a virologic cure.

### 3.1. Patient Demographics

Among the 59 patients who were initiated on DAA regimens, a majority were male (78%), were White/Non-Hispanic (50.8%), had HCV monoinfection (61%), were aged 50 years old or older (56%), and had Medicaid insurance (91.5%) (Table 1). Baseline characteristics were not statistically significantly different between groups. The majority of patients contracted HCV infection from intravenous drug use (*n* = 57, 96.6%). Two patients were unsure of how they acquired the infection.

**Figure 1:**
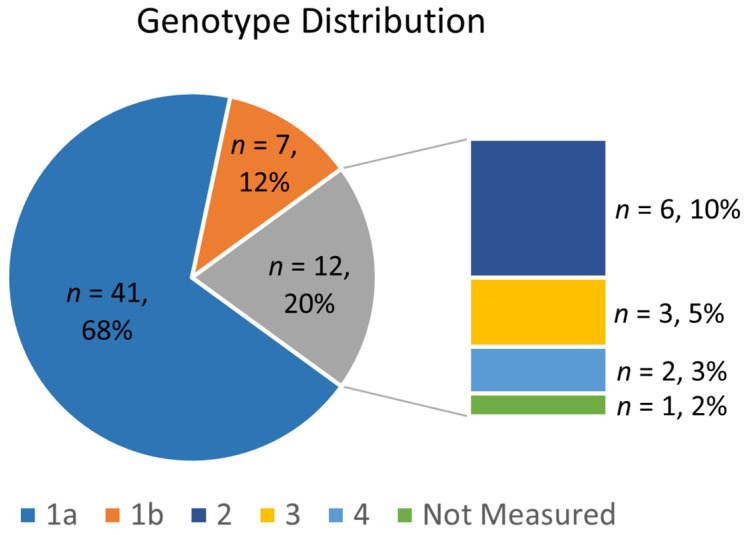
HCV genotype distribution of patients with HCV mono- and HIV co-infection.

The majority of patients had Fibrosure stages F1–F2 (*n* = 24, 40.7%), followed by F3–F4 (*n* = 19, 32.2%), and F0 (*n* = 14, 23.7%). Two patients received treatment without having a Fibrosure result within three months of treatment initiation (Figure 1). Patients who had moderate-to-severe liver fibrosis (F3–F4 stage) or advanced liver disease were placed on the sofosbuvir/velpatasvir (SOF/VEL) treatment approach to reduce the risk of worsening their liver condition, a problem that may be associated with glecaprevir/pibrentasvir (GLE/PIB) [15,16].

Patients’ HCV genotypes were also determined, where 68% had genotype 1a, 12% had 1b, 10% had genotype 2, and the rest had genotypes 3 and 4 (5% and 3%, respectively). One patient did not have their genotype available at the time of treatment initiation (Figure 1). This patient had HCV monoinfection and received GLE/PIB for their DAA treatment.

Patients received pan-genotypic DAA regimens that were active against all six HCV genotypes. They included two regimens, GLE/PIB and SOF/VEL. Forty-five patients (75%) received GLE/PIB for 8 weeks, and fourteen patients (23%) received SOF/VEL for 12 weeks. One patient was switched from the GLE/PIB regimen to the SOF/VEL regimen due to developing a rash; she completed the treatment and achieved SVR. Another patient with HCV/HIV co-infection and genotype 1a was placed on sofosbuvir/ledipasvir for treatment because it would not interact with his HIV antiretroviral regimen and was preferred over SOF/VEL on his health insurance’s drug formulary. His baseline HCV RNA viral load was 36,500 IU/mL.

### 3.2. Sustained Virologic Response

Twenty-one patients achieved SVR in the HCV monoinfected group (67.7%), and seventeen patients achieved SVR in the HCV/HIV co-infected group (73.9%). In the mITT analysis, the overall SVR rate among those who completed the treatment was 76.0%. SVR rates could not be measured for 10 patients with HCV monoinfection and five patients with HCV/HIV co-infection due to loss to follow-up care or loss of health insurance during the COVID-19 pandemic (Table 2).

## 4. Discussion

The pilot retrospective study conducted at AltaMed showed that a pharmacist-led HCV treatment program yielded results comparable to larger, real-world HCV studies with pharmacist involvement in FQHCs and academic institutions [9,11,12]. Hachey et al. examined the HCV treatment cascade and its impact at several FQHCs that served a large rural population in the western US. Their retrospective chart review from 1 January 2013 to 30 June 2016 showed that 389 patients were diagnosed with HCV monoinfection, 93 received DAA treatment, and 79.6% achieved SVR12 [9]. Our study demonstrated a similar SVR rate at 76%. Hachey and colleagues also addressed the multiple barriers to HCV treatment that rural patients face when seeking diagnosis confirmation, such as HCV testing, inability to pay for the medications and laboratory tests, provider knowledge, and access to treatment within an FQHC. They reported that the pharmacy team (pharmacists and interns) could help to lower the barriers to treatment access by helping patients enroll in Medicaid programs, completing manufacturer prescription assistance program paperwork, and processing prior authorization requests from private insurance companies [9]. While our study population is not rural, they face many of the same challenges and inequities related to access to care. The ambulatory care pharmacists were able to implement similar strategies to that proposed by Hachey and colleagues to ensure timely and efficient access to treatment.

Another study that assessed the impact of a pharmacist-led HCV drug management service in an FQHC setting was conducted by Downes et al. [11]. The observational study was conducted over four years at a Midwest FQHC with clinics located throughout Omaha, NE, USA. After the patients were screened and diagnosed with HCV infection, they were referred to one of two groups: usual care provided by a medical provider or by the pharmacist team. Two ambulatory care pharmacists worked under a protocol to initiate DAA treatment, document treatment status, assess their tolerability and safety, arrange laboratory testing, and schedule follow-up appointments [11]. Our study employed a similar workflow for pharmacist-driven care. In the study by Downes et al., there were 162 patients referred to the pharmacy team for treatment initiation, where the majority were 60 years and older, male, White, English-speaking, and uninsured. A total of 61 (37.7%) patients started treatment, with 4 patients not completing treatment because of loss to follow-up or loss of insurance coverage. In total, 57 patients completed treatment, and 45 of the 46 patients achieved SVR 12 weeks after treatment completion, which was 97.8% according to per-protocol analysis [11]. In contrast, our study included a greater proportion of individuals between the ages of 18 and 59 (74.6% of participants) and a larger proportion of individuals with Medicaid coverage (91.5% of participants). Despite differences in demographics between the two studies, we maintain a commendable SVR rate of 76%. While this is lower than the rate seen by Downes and colleagues, additional exploration is warranted to determine if other factors impact SVR achievement with more diverse populations and for a longer study period.

Koren et al. conducted a multicenter retrospective cohort study of patients receiving DAA treatment under the care of pharmacists. They examined the impact of a pharmacist-driven HCV treatment model at four large academic medical institutions in the Midwest from 1 January 2014 to 12 March 2018 [12]. A total of 1253 patients started treatment with 95 being lost to follow-up, and 24 patients discontinued treatment. Under their treatment protocol, 1079 patients achieved HCV virologic cure out of the 1134 patients who completed treatment (per-protocol SVR rate of 95.1%). The SVR rate was 86.1%, which was the intent-to-treat analysis rate when including patients lost to follow-up care and discontinued DAA treatment [12]. Koren et al. demonstrated that HCV treatment by clinical pharmacists in an open medical system network could lead to high SVR rates that were comparable to those by specialists and primary care physicians. Our study similarly demonstrated positive SVR rates with the pharmacist-directed treatment of HCV infection. Of note, in comparison with this and the previous studies described, we had a higher proportion of individuals with HCV/HIV co-infection.

Of the 67 patients referred to the pharmacist-led HCV treatment program at AltaMed, 88% started DAA treatment, 85% completed their DAA treatments, and 76% achieved SVR12 based on mITT analysis. The one patient with HCV monoinfection and whose HCV genotype could not be measured at the time of treatment initiation received GLE/PIB for their DAA regimen due to their preference for completing the treatment in eight weeks and a major drug–drug interaction with their concomitant medication, omeprazole, if SOF/VEL was prescribed. When our HCV treatment program was implemented in 2019, there were more patients referred for treatment initiation and management compared to patients referred in 2020 and 2021. Prior to the COVID-19 pandemic, the HCV patients in the program were scheduled for monthly appointments with the pharmacists for treatment adherence re-assessment and HCV RNA viral load test follow-up. The standard monthly HCV RNA tests were based on the older AASLD treatment guidance for closer monitoring of treatment response [17,18]. The HCV RNA tests were ordered by the pharmacists, obtained by the nurses in the clinic, and sent out to nearby Quest Diagnostics or LabCorp facilities for analyses. However, in 2020 and during the pandemic, we had to reduce the frequency of HCV laboratory monitoring for the patients to three measurement time points: initial or baseline HCV laboratory results, the time of DAA treatment completion, and three months after treatment completion to determine SVR. Recent studies have reported that standard monthly HCV RNA monitoring may not be necessary. These studies were conducted in several countries including Rwanda, Brazil, South Africa, Thailand, Uganda, and the US [19,20]. They demonstrated that the favorable safety and tolerability and highly effective profiles of the oral DAA regimens could lead to reduced laboratory HCV RNA viral load monitoring and still achieve high SVR outcomes.

Not surprisingly, the patient referral rate to our treatment program declined markedly during the pandemic between 2020 and 2021. The pandemic adversely impacted the patients’ in-person clinic visits. Like almost all healthcare providers during the pandemic, we resorted to using telehealth as the main tool to initiate DAA regimens for newly referred patients with HCV infection and continued monitoring patients until they completed their DAA treatments. Overall, the limitations of our study included its small sample size, the shortened study time period, the retrospective nature of the pilot study, its single-center nature, and the impact of the pandemic on patient referrals and visits.

Despite these limitations, our program’s HCV treatment success rate (mITT SVR result of 76%) is respectable and could be compared to the SVR results of the above-mentioned HCV studies in an FQHC setting [9,10,11]. Of note, our study stood out from the comparative studies because we had more patients with HCV/HIV co-infection and potential for drug–drug interaction issues, while more were of Hispanic ethnicity and were within an age group younger than the “baby boomer” group (born between 1945 and 1965). In conclusion, this study has allowed us to explore the feasibility of a pharmacist-led HCV treatment program in a more diverse population than what has previously been reported in the literature. Additional studies are warranted to further investigate long-term outcomes, impacts on patient access to care, and the use of technology by pharmacists to provide clinical services post-COVID.

## Figures and Tables

**Table 1:** Baseline characteristics of patients who received DAA treatment.

	HCV Monoinfection	HCV/HIV Co-Infection	Total (*n* = 59)
**Sex**			
Male	25 (42.4%)	21 (35.6%)	46 (78.0%)
Female	11 (18.6%)	2 (3.39%)	13 (22.0%)
**Age (years)**			
18–29	4 (6.78%)	1 (1.69%)	5 (8.47%)
30–39	5 (8.47%)	5 (8.47%)	10 (16.9%)
40–49	7 (11.9%)	4 (6.78%)	11 (18.6%)
50–59	10 (16.9%)	8 (13.6%)	18 (30.5%)
≥60	10 (16.9%)	5 (8.47%)	15 (25.4%)
**Race/Ethnicity**			
White/Non-Hispanic	17 (28.8%)	13 (22.0%)	30 (50.8%)
White/Hispanic	13 (22.0%)	9 (15.3%)	22 (37.3%)
Black/Non-Hispanic	1 (1.69%)	1 (1.69%)	2 (3.39%)
Asian	0 (0%)	1 (1.69%)	1 (1.69%)
Not specified or undeclared	0 (0%)	4 (6.78%)	4 (6.78%)
**Health Insurance Type**			
Commercial	2 (3.39%)	0 (0%)	2 (3.39%)
Medicare	2 (3.39%)	1 (1.69%)	3 (5.08%)
Medicaid	34 (57.6%)	20 (33.9%)	54 (91.5%)

**Table 2:** Sustained virologic response (SVR) rate at 12 weeks after treatment completion.

	HCV Monoinfection	HCV/HIV Co-Infection	Total
HCV Treatment Regimens Initiated	36 (60.0%)	24 (40.0%)	60 *
HCV Treatment Completion	30 (60.0%)	20 (40.0%)	50
Patients who achieved SVR12	21 (55.3%)	17 (44.7%)	38
Treatment Failure	0 (0%)	1 (100%)	1
Not Measured	10 (66.7%)	5 (33.3%)	15
SVR12 Rate ^†^	67.74%	73.91%	76.0% ^‡^

* Represents the number of treatment regimens. One patient received two treatment regimens due to treatment failure. ^†^ Excludes patients with SVR12 dates outside of this study’s time frame. ^‡^ Overall SVR rate, mITT.

## Data Availability

The raw data supporting the conclusions of this article will be made available by the authors on request.

## References

[1] Thompson W.W., Symum H., Sandul A., Gupta N., Patel P., Nelson N., Mermin J., Wester C. (2022). Vital Signs: Hepatitis C Treatment Among Insured Adults–United States, 2019–2020. MMWR Morb. Mortal. Wkly. Rep..

[2] Ryerson A.B., Schille S., Barker L.K., Kupronis B.A., Wester C. (2020). Vital Signs: Newly Reported Acute and Chronic Hepatitis C Cases–United States, 2009–2018. MMWR Morb. Mortal. Wkly. Rep..

[3] Centers for Disease Control and Prevention (2022). Viral Hepatitis Surveillance–United States, 2020.

[4] Hepatitis C., World Health Organization Published 24 June 2022. https://www.who.int/news-room/fact-sheets/detail/hepatitis-c.

[5] Bhamidimarri K.R., Satapathy S.K., Martin P. (2017). Hepatitis C Virus and Liver Transplantation. Gastroenterol. Hepatol..

[6] U.S. Department of Health and Human Services (2020). Viral Hepatitis National Strategic Plan for the United States: A Roadmap to Elimination (2021–2025).

[7] Nguyen V.H., Kam L., Yeo Y.H., Huang D.Q., Henry L., Cheung R., Nguyen M.H. (2022). Characteristics and Treatment Rate of Patients with Hepatitis C Virus Infection in the Direct-Acting Antiviral Era and during the COVID-19 Pandemic in the United States. JAMA Netw Open..

[8] Mendizabal M., Alonso C., Silva M.O. (2019). Overcoming barriers to hepatitis C elimination. Frontline Gastroenterol..

[9] Hachey D.M., Holmes J.T., Aubuchon-Endsley N.L. (2020). Hepatitis C Treatment Cascade in a Federally Qualified Health Center. J. Community Health.

[10] Assoumou S.A., Wang J., Nolen S., Yazdi G.E., Mayer K.H., Puro J., Salomon J.A., Linas B.P. (2020). HCV Testing and Treatment in a National Sample of US Federally Qualified Health Centers during the Opioid Epidemic. J. Gen. Intern. Med..

[11] Downes J.M., Donovan A., McAdam-Marx C. (2022). Pharmacist-led drug therapy management for hepatitis C at a federally qualified health care center. J. Am. Pharm. Assoc..

[12] Koren D.E., Zuckerman A., Teply R., Nabulsi N.A., Lee T.A., Martin M.T. (2019). Expanding Hepatitis C Virus Care and Cure: National Experience Using a Clinical Pharmacist-Driven Model. Open Forum Infect. Dis..

[13] Bhattacharya D., Aronsohn A., Price J., Lo Re V., AASLD-IDSA HCV Guidance Panel (2023). Hepatitis C Guidance 2023 Update: AASLD-IDSA Recommendations for Testing, Managing, and Treating Hepatitis C Virus Infection. Clin. Infect. Dis.

[14] Chou R., Wasson N. (2013). Blood Tests to Diagnose Fibrosis or Cirrhosis in Patients With Chronic Hepatitis C Virus Infection. Ann. Intern. Med..

[15] FDA Drug Safety Communication. https://www.fda.gov/drugs/drug-safety-and-availability/fda-warns-about-rare-occurrence-serious-liver-injury-use-hepatitis-c-medicines-mavyret-zepatier-and.

[16] (2023). Mavyret Prescribing Information.

[17] AASLD/IDSA HCV Guidance Panel (2015). Hepatitis C guidance: AASLD-IDSA recommendations for testing, managing, and treating adults infected with hepatitis C virus. Hepatology.

[18] Morris L., Selvey L., Williams O., Gilks C., Kvassy A., Smirnov A. (2020). Hepatitis C cascade of care at an integrated community facility for people who inject drugs. J. Subst. Abuse Treat..

[19] Grant P., Shumbusho F., Van Nuil J.I., Kateera F., Mukherjee J., Kabahizi J., Ntaganda F., Nsanzimana S., Mbituyumuremyi A., Damascene M.J. (2020). Safety and Efficacy of Limited Laboratory Monitoring for Hepatitis C Treatment: A Blinded Clinical Trial in Rwanda. Hepatol. Commun..

[20] Solomon S.S., Wagner-Cardoso S., Smeaton L., Sowah L.A., Wimbish C., Robbins G., Brates I., Scello C., Son A., Avihingsanon A. (2022). A minimal monitoring approach for the treatment of hepatitis C virus infection (ACTG A5360 [MINMON]): A phase 4, open-label, single-arm trial. Lancet Gastroenterol. Hepatol..

